# Risk of colorectal adenomas and cancer in monoallelic carriers of *MUTYH* pathogenic variants: a single-centre experience

**DOI:** 10.1007/s00384-021-03983-x

**Published:** 2021-07-09

**Authors:** R. Patel, P. McGinty, V. Cuthill, M. Hawkins, S. K. Clark, A. Latchford

**Affiliations:** 1 Polyposis Registry, St Mark’s Hospital, Harrow, UK; 2 Department of Surgery and Cancer, Imperial College London, London, UK

**Keywords:** MAP, *MYH* associated polyposis, *MUTYH* associated polyposis, Hereditary gastrointestinal cancer

## Abstract

**Purpose:**

The carrier frequency of *MUTYH* pathogenic variants in the population may be as high as one in 45. Some studies have found an increased risk of colorectal cancer (CRC) in monoallelic carriers of *MUTYH* pathogenic variants, but the role of early surveillance colonoscopy is not conclusive. This study aimed to assess the outcomes of colonoscopy surveillance in *MUTYH* carriers.

**Methods:**

Patients, with a monoallelic pathogenic variant in *MUTYH*, found at cascade testing, were identified from the St Mark’s Hospital Polyposis Registry database. Findings at surveillance colonoscopy were reviewed.

**Results:**

Two hundred and forty-nine carriers were identified, of whom 125 had undergone at least one surveillance colonoscopy. Twenty-eight patients (22%) developed at least one adenoma; all adenomas had low-grade dysplasia (LGD). The median age at first colonoscopy was 36 years (range 16–75 years). The median age at first adenoma detection was 43 years (range 22–75 years). The cumulative incidence of adenoma development by age 30, 40, 50, 60 and 70 years was 3.2%, 8.8%, 15.2%, 18.4% and 20.8%, respectively. No CRCs were observed.

**Conclusions:**

Our cohort of monoallelic carriers of *MUTYH* pathogenic variants is a relatively younger group than adults entering population screening colonoscopy, but a high adenoma rate was not observed. No CRCs were detected, suggesting that current guidance that these individuals should be managed in the same way as the general population is reasonable.

## Background

Inheritance of a pathogenic variant of *MUTYH* from each parent leads to *MUTYH*-associated adenomatous polyposis (MAP) (autosomal recessive inheritance) [1, 2]. Controversy exists as to whether “carriers” of a single pathogenic variant are at an increased risk of development of colorectal adenomas and cancer.

*MUTYH* is a base excision repair gene; the protein product works within a system [3] to ensure that G:C pairing in the genome remains intact. Oxidative stress from environmental factors can cause guanine to become oxidised to 8-oxo-G. This leads to an incorrect T:A pairing instead of G:C during replication [4], which is rectified by the MUTYH protein. Somatic G:C to T:A transversions have been identified in *APC* and *KRA*S genes from adenomatous polyps in patients with MAP [5]. In addition, hyperplastic and serrated sessile serrated polyps (which also occur with increased frequency in MAP) have been found to contain G:C to T:A transversions in the *KRAS* gene [6].

The carrier frequency of *MUTYH* pathogenic variants in the population may be as high as 1:45 [7]. The common European pathogenic variants are Tyr179Cys and Gly396Asp; Glu480Ter mutation is most commonly found in individuals from the Indian subcontinent [8].

The management of patients who are monoallelic pathogenic variant carriers is open to debate. Some studies have suggested that carriers may be at increased risk of the development of colorectal cancer (CRC) [9–11]. Most, however, have not revealed a significantly increased risk [12, 13], and most guidelines currently do not advocate screening other than national bowel cancer screening programmes [3]. The management of these patients has been constantly evolving over time as we improve our knowledge of the risk that these individuals carry. We previously reported a higher adenoma detection rate than expected in *MUTYH* carriers [14]. On this basis, in our institution, monoallelic carriers were offered colonoscopy every 5 years from age 35, later modified to commence from age 40 years, until entry into the national bowel cancer screening programme.

This retrospective study aimed to assess the outcomes of colonoscopy surveillance in *MUTYH* carriers.

## Methods

**Fig. 1:**
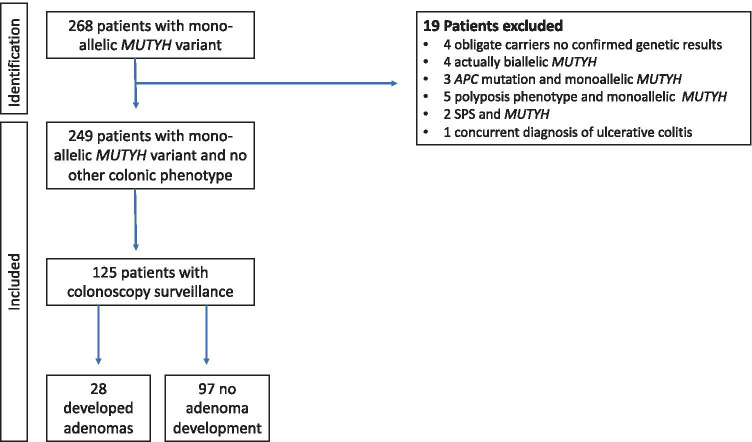
Flow chart of individuals identified by cascade testing that were included in study and who underwent surveillance colonoscopy

The St Mark’s Hospital Polyposis Registry database was used to identify patients with confirmed monoallelic *MUTYH* pathogenic variants. Only patients following cascade testing and confirmed genetic results were included; obligate carriers without confirmed genetic results were excluded (*n* = 4). Patients presenting clinically were excluded; these included three with a co-existing *APC* pathogenic variant, five with a polyposis phenotype (all had more than 50 to 100 polyps), two with coexisting serrated polyposis, and one with a concurrent diagnosis of ulcerative colitis (Fig. 1).

The following data were obtained: gender, pedigree, details of monoallelic *MUTYH* pathogenic variant and types of investigations/surveillance performed. Surveillance data were obtained between 1994 and 2019. In our centre, surveillance colonoscopy was introduced following the discovery of the *MUTYH* gene in 2003 and performed every 5 years from 2004 to 2018. Prior to 2003, surveillance was conducted for patients who had first-degree relatives with polyposis and an unidentified germline pathogenic variant; those subsequently found to carry a variant in *MUTYH* were included in this study. The age of commencement of 5 yearly surveillance changed during this period, from age 35 from 2003 and 40 years from 2013 to 2018. Surveillance endoscopy findings including adenoma development, the number of polypectomies and pathology results were also analysed.

Patients receiving care outside of our centre were only included in this study if copies of endoscopy and pathology reports were available for review.

### Statistical analysis

Stata v.15.1 (StataCorp LLC, College Station, Texas, USA) was used for statistical analysis. Kaplan–Meier survival analysis and cumulative incidence were calculated for adenoma development. Mann–Whitney U, chi^2^ and Fisher’s exact tests were used to calculate demographic differences between patients with and without adenoma detection during surveillance. A p-value of < 0.05 was used as a threshold to suggest a statistically significant difference between variables.

## Results

Two hundred and forty-nine confirmed monoallelic pathogenic variant carriers (120 female, 129 male) found by cascade testing from 73 families were identified from the St Mark’s Hospital Polyposis Registry (see Fig. 1). One hundred and twenty-five individuals (50%) had colonoscopy surveillance.

There were a total of 288 investigations: 232 colonoscopies in 125 patients (median 1, range 1–7), 54 flexible sigmoidoscopies in 29 patients and two CT colonographies in one patient. Thirty-five of 232 colonoscopies (15%) were performed between 1994 and 2002; the remainder was from the year 2003.

The colonoscopy data were examined to determine adenoma detection in this group of patients. Demographic differences between the groups are shown in Table 1. The median age at first colonoscopy was 36 years (range 16 to 75).

**Table 1:** A table of demographics to show differences between patients who had adenomas detected on surveillance and those that did not have adenomas detected. MWU = Mann–Whitney U test

	Adenoma	No adenoma	P Value
Number of individuals	28	97	
Median number of coloscopies during surveillance period	3 (range 1 to 7)	1 (range 1 to 5)	0.007 (MWU)
Median age at last surveillance	48 (22 to 75)	35 (range 18 to 69)	< 0.05 (MWU)
Gender			Chi^2^ = 0.167
Male	18	48	
Female	10	49	
Genetic group			0.134 (Fisher’s exact)
Glu480Ter	19	64	
Tyr179Cys	3	10	
Gly396Asp	4	3	
Tyr104Ter	1	6	
Other	1	14	

One hundred and thirty colonoscopies were performed in 73 patients before the age of 40 years, 67 colonoscopies were performed in the age group 40 to 55 years and 35 colonoscopies were performed in patients over 55 years of age. The reason for a colonoscopy before 40 years of age in the majority of patients was because of the younger start of screening (at 35 years) early on in our programme. However, the exception to this was the presence of symptoms (change in bowel habit, bleeding or anaemia) in five cases, or because the family pathogenic variant had not been identified at the time of that particular surveillance colonoscopy (in 14 cases). Of the five patients who had a colonoscopy for symptoms and were under 40 years of age, only one patient was found to have an adenoma at the age of 38; the lesion was a 3-mm adenoma with low-grade dysplasia (LGD). Of the 14 patients who had a colonoscopy under 40 years of age (due to a pathogenic variant not being identified at the time of surveillance), two were found to have a small adenoma (1–4 mm) with LGD at ages 21 and 33, respectively.

Eighteen individuals were found to have a non-adenomatous lesion (hyperplastic, fibro-epithelial and inflammatory). Ninety-seven individuals were never found to have an adenoma on colonoscopy (Table 2); 28 developed at least one adenoma (22%). The median age at first adenoma detection was 43 years (range 22 to 75). The cumulative number of adenomas detected is detailed in Table 2. One patient developed a cumulative adenoma count of over 10. The group found to have had adenomas were significantly older at last surveillance colonoscopy compared to the group who had not developed adenomas (*P* < 0.05).

**Table 2:** A table including the 125 individuals who had a surveillance colonoscopy and their cumulative adenoma count during follow-up

Cumulative adenoma number	Number of individuals
0	97
1	8
2	9
3	5
4	1
6	2
7	1
8	1
17	1
Total	125

**Fig. 2:**
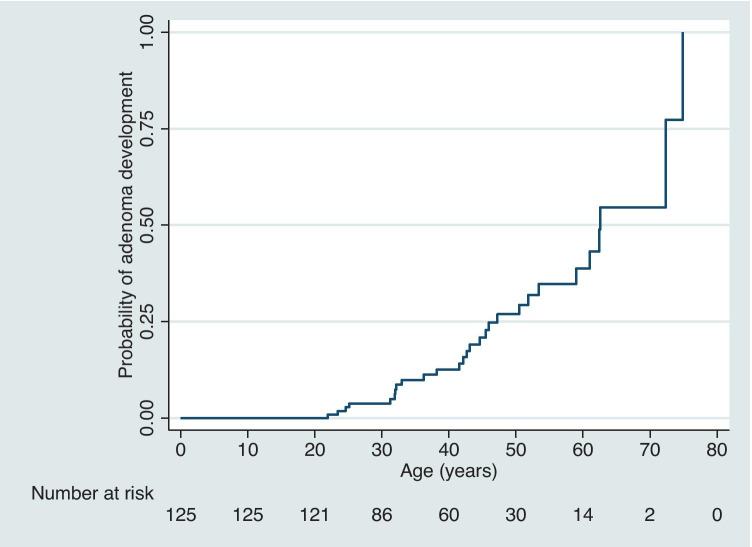
Kaplan–Meier survival curve for age of progression to first adenoma detection in patients undergoing colonoscopy

The cumulative incidence of adenoma development by age 30, 40, 50, 60 and 70 years was 3.2%, 8.8%, 15.2%, 18.4% and 20.8%, respectively. Kaplan–Meier survival analysis of patients who had surveillance estimated that by 50 years of age a quarter had developed at least one adenoma (Fig. 2). The majority of patients did not develop advanced adenomatous lesions (defined as high-grade dysplasia (HGD), villous adenoma or adenoma size > 10 mm) (Table 3). Indeed, only one patient developed an advanced adenoma at the age of 72; this individual was the parent of a patient with MAP and surveillance started at the age of 72. They had a cumulative polyp count of 17, with one lesion being larger than 10 mm, with villous features on histology. Neither gender nor type of monoallelic *MUTYH* pathogenic variant was associated with adenoma development.

**Table 3:** A table of the 28 patients who developed adenomas to summarise characteristics of adenoma detected (most advanced adenoma histology, dysplasia and largest size)

	Number of patients	%
Most advanced histology		
TA	25	89
TVA	2	7
VA	1	4
Total	28	
Degree of dysplasia		
LGD	28	100
HGD	0	
Cancer	0	
Total	28	
Largest size		
1–4 mm	18	64
5–9 mm	9	32
≥ 10 mm	1	4
Total	28	

## Discussion

Our single-centre retrospective study of monoallelic *MUTYH* pathogenic variant carriers from a Registry database did not observe any case of CRC. In addition, adenoma detection in 22% of the group seemed similar to that of general population cohorts [14–16]. The median age of adenoma detection in this cohort was 43 years. Twenty-two percent of our carrier cohort under surveillance developed at least one adenoma. This suggests that the risk of adenoma development may be similar to that of the general population; a large cohort study of 20 792 colonoscopies for any indication (but surveillance excluded) found that 24.6% of all patients aged over 50 had at least one adenoma [14]. A further population-based study of 12 574 patients aged from 55 to 64 years old undergoing screening colonoscopy found at least one adenoma in 30.7% of patients [15]. In a screening population of 1256 patients (aged 50 to 75 years), 21% of patients were found to have non-advanced lesions (LGD, < 10 mm and tubular-adenomatous/ tubulo-villous lesions), 9% of patients were found to have advanced lesions (HGD/ > 10 mm/villous architecture) and 0.6% had cancer [16]. Our study lacks a control arm for comparison of progression to development of adenomas; however, obtaining control data for this type of cohort is challenging given that a “healthy” population is unlikely to undergo screening surveillance at a comparably young age.

Despite this, and our study being smaller and of a slightly younger group of *MUTYH* carriers compared to screening studies [14–16], the percentages of patients with adenomatous lesions do seem similar. However, the fact that only one patient developed an advanced adenoma (lesion that was > 10 mm with LGD), which was detected after surveillance started at the age of 72 years is reassuring. There were no cases of CRC in our group of monoallelic *MUTYH* mutation carriers undergoing surveillance colonoscopy. This cohort was identified through cascade testing and thus represents a much younger cohort compared to some other studies of such individuals. Since *MUTYH* was discovered in 2003, prior to this all individuals with a first-degree relative with polyposis would undergo regular surveillance.

Some studies have suggested that there is an increased risk of CRC in monoallelic carriers. A retrospective study of 347 parents (assumed obligate carriers) of unrelated MAP index cases was found to have a two-fold increase in colorectal risk [10]. Since that study was looking at the parents of MAP index cases, they were an older cohort (mean age 70 years) who had not previously undergone surveillance, which is different from our younger surveillance cohort and thus may explain the increased cancer risk observed.

A more recent large collaborative study of 264 families from the USA, Canada, Australia and New Zealand found that monoallelic mutation carriers had on average a 2.5-fold increased risk of CRC compared to the general population [9] at 50 years of age the cumulative risk was low (0.8%). A further case–control study of 120 patients with CRC compared to healthy controls found that the variant Tyr179Cys was associated with an increased risk of CRC compared to controls (12.5% vs 4%) [17] (Gly396Asp was not associated with an increased risk and other variants were not tested). Another study characterising *MUTYH* variants in Ashkenazi Jews compared to individuals of other ancestry found that, although monoallelic *MUTYH* mutations were rare, they did find that, when present, they were significantly associated with a personal history of CRC regardless of ancestry (OR 1.78; 95% CI 1.21–2.49; P < 0.01) [18].

Our study did not detect any genotype–phenotype associations with respect to adenoma development in this carrier cohort. A Canadian population-based series of 1238 colorectal cancer patients identified 29 monoallelic mutation carriers [19]. The authors found that there was an association between Tyr179Cys and Gly396Asp carriers and increased colorectal cancer risk.

A large proportion of our monoallelic *MUTYH* pathogenic variant carriers are of Indian Gujerati descent (66% of our cohort had a Glu480Ter mutation); thus, it may be that the Glu480Ter mutation has a different phenotypic affect to pathogenic variants found typically in Caucasian populations. Larger collaborative studies are required to explore this.

A weakness of our study of carriers is that the majority of our patients had only had a single surveillance colonoscopy at a relatively young age. In addition, polypectomy may have prevented advanced neoplasia. Thus, our data cannot conclusively demonstrate that this population is not at a slightly increased risk, and larger longer-term follow-up data are required to determine colorectal risk in this group of patients.

## Conclusion

This cohort of cascade-tested individuals on surveillance found no cases of CRC, and only one case of advanced adenoma. Our data support current UK guidance that individuals carrying monoallelic *MUTYH* variants should be managed in the same way as the general population [3].

## Data Availability

Due to patient confidentiality and GDPR, data will not be publicly available. Reasonable requests for de-identified data should be made to andrew.latchford@nhs.net.
